# Multi-Physically Cross-Linked Hydrogels for Flexible Sensors with High Strength and Self-Healing Properties

**DOI:** 10.3390/polym15183748

**Published:** 2023-09-13

**Authors:** Yulin Zhang, Shiyu Wang, Yi Tian, Long Chen, Yuhan Du, Gehong Su, Yu Hu

**Affiliations:** 1 School of New Energy Materials and Chemistry, Leshan Normal University, Leshan 614000, China; zhangyulin_76rc@wx.lsnu.edu.cn (Y.Z.);; 2 Leshan West Silicon Materials Photovoltaic and New Energy Industry Technology Research Institute, Leshan 614000, China; 3 College of Science, Sichuan Agricultural University, Xin Kang Road, Yucheng District, Ya’an 625014, China; 4 State Key Laboratory of Molecular Engineering of Polymers, Fudan University, Shanghai 200433, China; 5 Material Corrosion and Protection Key Laboratory of Sichuan Province, Zigong 643000, China

**Keywords:** physical hydrogel, hydrophobic association, hydrogen bonding, ionic complexation, sensor

## Abstract

Excellent mechanical properties and self-healing properties are very important for the practical application of hydrogel flexible sensors. In this study, acrylic acid and stearyl methyl acrylate were selected as monomers to synthesize hydrophobic association hydrogels, and multi-physically cross-linked hydrogels were synthesized by adding ferric chloride and polyvinyl alcohol to introduce ion interaction and a hydrogen bond cross-linking network. The hydrogels were characterized by FTIR, XRD and SEM, and the mechanical properties and self-healing properties were tested using a universal testing machine. It was confirmed that the strength of the hydrogel was significantly improved with the addition of ferric chloride and polyvinyl alcohol, and the hydrogel still showed good self-healing properties. Further testing of its application as a conductive sensor has demonstrated sensitive and stable motion sensing capabilities. This provides an important reference for high-performance hydrogel sensors with both high strength and self-healing properties.

## 1. Introduction

As flexible materials with a high water content, hydrogels have been the subject of extensive research in a number of areas, such as drug release [1,2], bioengineering [3,4], supercapacitors [5], desalination [6,7] and agriculture [8]. In the last decade, the research of flexible materials, especially hydrogels, has developed rapidly in the fields of electronic skins [9], soft robots [10], smart interactive devices [11], and wearable devices [12,13]. The demand for flexible sensors with good strength, elongation, self-healing, stability, and other properties has increased rapidly. As a material with the potential to meet the above requirements, there have been many reports on conductive hydrogels as ideal material candidates for flexible sensors [14,15,16,17]. To meet the performance requirements for flexible sensor materials, hydrogels must possess some specific properties, such as good conductive sensing properties, mechanical properties, self-healing properties, biocompatibility [18,19,20]. The conductivity of hydrogels is usually achieved by various free-moving ions and conductive fillers in the gel network, such as polyaniline, graphene, MXenes [21,22,23]. When a hydrogel is subjected to external forces, the shape of the hydrogel, such as cross-sectional area and length, is deformed accordingly, resulting in a corresponding change in the overall resistivity of the hydrogel, and thus hydrogels can be used as sensors to detect changes in tension, pressure, limb motion, object motion and vocal cord vibration [24,25].

According to the mode of cross-linked structure, hydrogels could be classified into chemically cross-linked hydrogels and physically cross-linked hydrogels [26,27]. Among them, chemically cross-linked hydrogels are generally formed by free radical polymerization, graft copolymerization, water soluble polymer cross-linking and other cross-linking polymerization reactions, and their cross-linking network is composed of a covalent bond. Therefore, chemically cross-linked hydrogels usually have a stable cross-linked network structure, and exhibit high strength, good stability and fatigue resistance. However, the cross-linked network is generally not reversible, and it cannot be restored by itself after being destroyed. In contrast, the network of physically cross-linked hydrogels is formed by non-covalent cross-linking sites such as hydrogen bonds, hydrophobic bonds, and ionic interactions [28,29]. The physically cross-linked network structure is, to a certain extent, revertible, so most of the physically cross-linked hydrogels exhibit good self-healing and reusability [30,31,32], and the physical cross-linked hydrogels do not use chemical cross-linking agents, which is conducive to reducing the toxicity of the system. Therefore, based on the above advantages, various physically cross-linked hydrogel systems have attracted the attention of researchers in recent years. However, due to the lack of covalently bonded cross-linked structures, the network structures of physically cross-linked hydrogels are often not stable enough, resulting in poor swelling stability, easy dissolution upon prolonged immersion in water, and low macroscopic strength, making it difficult to meet the practical requirement [33,34]. In order to improve the stability of both the chemically and physically cross-linked hydrogels and increase the mechanical strength, many researchers have described various strategies to synthesize high-strength gels, such as nanocomposite hydrogels [35,36], interpenetrating hydrogels [37] and dual-network hydrogels [38,39]. Unfortunately, the self-healing properties and the mechanical strength are often mutually limiting for hydrogels, with higher cross-link density leading to higher stability and strength, but hindering the movement and rearrangement of molecular chains, leading to reduced self-healing properties [40].

The strategy of this study is to select suitable physically cross-linked gel systems and synthesize multiple physically cross-linked hydrogels. As a typical physically cross-linked hydrogel, hydrophobic association hydrogels (HA gels) usually show good self-healing ability, have a relatively loose network structure and good plasticity, and can swell up to twenty times their original size [41,42]. However, the simple HA gel network was less stable and would dissolve after prolonged immersion in water [41,42,43]. Polyvinyl alcohol (PVA) is a water-soluble polymer that can form a physically cross-linked network of hydrogen-bonded crystalline cross-linked clusters in aqueous solution by simple freeze–thaw treatment [44,45,46]. Cross-linking with multivalent metal ions is also an effective way to improve the mechanical properties of hydrogels and impart self-healing properties [47,48,49]. The synthesis of dual or multiple physically cross-linked hydrogels was a feasible strategy to simultaneously improve the strength and self-healing properties of hydrogels, e.g., Jingdi Chen et al. used sodium alginate, chitosan, and zinc ions to prepare high-strength and non-biotoxic double physically cross-linked hydrogels for wound dressings [34], and Jieshan Qiu et al. used PVA, carboxymethyl cellulose, and zinc ions to prepare high-strength physically cross-linked hydrogels for the preparation of supercapacitors [47]. There have been many reports on the synthesis of hydrogels with dual or multiple physical cross-linking structures, and very good results were achieved in the synthesis of high-strength hydrogels, but these articles mainly focused on multiple hydrogen-bonded cross-linking structures or the combination of hydrogen-bonded cross-linking structures and ionic interactions, which may lack cross-linking interactions and material selectivity. In this paper, multiple physical cross-linking structures combine hydrophobic association, hydrogen bonding and ionic cross-linking to enrich the selectivity of cross-linking structures, which brings the possibility of achieving more functionalities. Therefore, in this study, using acrylic acid as the hydrophilic monomer, HAPAA (hydrophobic association polyacrylic acid) gels were synthesized as the gel matrix. Ferric chloride and PVA were added to the system, where the Fe^3+^ and carboxylate ions formed ionic cross-linking interactions, resulting in a physically double cross-linked hydrogel consisting of hydrophobic cross-linking and ionic cross-linking. The linear PVA chains formed a semi-interpenetrating (SI) network structure with the HAPAA hydrogel network, and extensive hydrogen-bonding interactions between the large number of carboxyl groups on the HAPAA network and hydroxyl groups on the PVA chains improved the stability of the hydrogel system. Finally, the PVA chains formed a crystalline cross-linked network through freeze–thaw treatment, resulting in a composite network hydrogel with multiple physically cross-linked networks.

The cross-linked network of this hydrogel system consists of three physically cross-linked structures, namely hydrophobic association, ionic complex and hydrogen bonding, so that the hydrogel has both good self-healing and good mechanical properties. In this study, the gels were first characterized by a Fourier transform infrared spectrometer (FTIR), X-ray diffraction analysis (XRD), and scanning electron microscopy (SEM) to determine the successful synthesis of this multi-physically cross-linked hydrogel. Then, in order to quantify the various properties of this hydrogel, the mechanical properties, swelling properties, energy dissipation capacity, and self-healing properties of this hydrogel were tested. Finally, the potential applications as conductive sensors were further tested and provided an important reference for the fabrication of high-performance gel sensors.

## 2. Materials and Methods

### 2.1. Materials

Acrylic acid (AA), stearyl methyl acrylate (SMA), Sodium dodecyl benzene sulfonate (SDBS) and potassium persulfate (KPS) were purchased from Aladdin Reagent (Shanghai, China). Ferric chloride hexahydrate (FeCl_3_·6H_2_O) and PVA 1799 were purchased from Adamas Reagent (Shanghai, China). The DI water was prepared using a reverse osmosis membrane water purifier (Sichuan Water Purifier WP-2RO-300L, Chengdu, China).

### 2.2. Preparation of Multi-Physically Cross-Linked Hydrogels

The dual network hydrogels were prepared using a two-step process. In the first step, HAPAA hydrogels containing ferric chloride and chain PVA pre-dissolved in DI water were obtained by one-pot thermal polymerization. In the second step, the chain PVA was formed into PVA crystalline cross-linked network by three freeze–thaw treatments. The details of the process are as follows: (1) appropriate amounts of PVA and DI water were dispersed in a round-bottomed flask and underwent vigorous stirring in an oil bath at 90 °C for 3 h to obtain a homogeneous solution. The solution was then removed from the oil bath and cooled to room temperature. (2) Appropriate amounts of AA (hydrophilic monomer), SDBS (surfactant), SMA (hydrophobic monomer), FeCl_3_·6H_2_O (Fe^3+^ supply) were added to the PVA solution and stirred to homogeneity. (3) KPS initiator was added to the mixed solution and bubbled with nitrogen for 5 min. The mixed solution was then transferred to glass molds. (4) The glass molds were placed in a water bath at 60 °C for 12 h to obtain the hydrophobic association hydrogel network. (5) Then, parts of the hydrogels were frozen at −15 °C for at least 5 h, then left at room temperature for more than 1 day. This was repeated three times to obtain dual network hydrogels. The prepared hydrogels were labeled as SI-X-Y or DN-X-Y, where SI to referred semi-interpenetrating hydrogel without freeze–thaw treatment, and DN referred to dual network hydrogel after freeze–thaw treatment. X represented the weight percentage of PVA in the whole system, and Y represented the molar percentage of ferric chloride hexahydrate compared with acrylic acid. The hydrogel synthesis formula is shown in Table 1.

### 2.3. FTIR Spectroscopy

FTIR spectroscopy was performed using a Nicolet iS50 (Thermo Fisher, Walatham, MA, USA). The gels were placed in an oven at 40 °C and dried to constant weight to obtain dry samples, which were then ground and characterized using the potassium bromide press method, measuring wave numbers in the range of 400–4000 cm^−1^ with a resolution set at 2 cm^−1^ and repeating the scan for 16 times.

### 2.4. X-ray Diffraction Analysis

The hydrogel samples were dried in a 40 °C oven to obtain dried small rectangular slices, which were then analyzed using a D/MAX 2400 X-ray diffractometer (RIGAKU, Tokyo, Japan) with target Cu Kα (λ = 0.15406 nm). The power was 12 kW, the scanning speed was 4° min^−1^ and the scanning range was 5–60°.

### 2.5. Scanning Electron Microscopy

The gel was cut into thin slices of approximately 3 mm in height, and the hydrogel was quenched with liquid nitrogen. The samples with good cross section were selected and placed in a Petri dish and put into a freeze-drying machine (YTLG-10A, Shanghai, China). The temperature of the cold trap was set to −60 °C, and the vacuum level was set to 2 Pa. After 2 days of lyophilization, the samples after platinum spray treatment were analyzed using a CIQTEK SEM 5000 microscope (CIQTEK, Hefei, China).

### 2.6. Rheological Properties

The hydrogels were cut into thin slices of approximately 10 mm diameter and 5 mm height, then the slices were subjected to rheological tests using a rheometer (TA AR2000ex, New Castle, DE, USA). The test temperature was set at 25 °C and the shear strain was set at 1%.

### 2.7. Uniaxial Tensile Tests

The tensile test apparatus is a INSTRON5567 universal testing machine (Instron, Norwood, MA, USA) with the load of 1 KN; we set a pull-up speed of 100 mm/min with a distance of 20 mm for the uniform speed stretching gel until the gel ruptured, in order to obtain the tensile stress–strain data of hydrogel.

### 2.8. Energy Dissipation Performance

Using the same experimental conditions as uniaxial stretching for cyclic stretching test, the hydrogel specimen was stretched to the maximum elongation of 500% and then returned to its original length, and the changes of its stress–strain curve were recorded.

### 2.9. Swelling Performance Test

A small piece of each component gel was cut off and dried to obtain a dry weight *Wd*. The dried sample was then placed in a beaker filled with DI water and the sample was removed at regular intervals and the surface water was gently absorbed with filter paper and weighed to obtain *Ws* until the swelling equilibrium was reached or the gel structure collapsed, and the swelling ratio was calculated using the following formula:

$$Swelling\;Ratio=\frac{Ws}{Wd}-1$$

The swelling behaviors were evaluated by Schott’s second-order swelling kinetics model. $w_{\infty }$ is the gel mass at equilibrium swelling, t is the swelling time, and w is the gel mass corresponding to the swelling time.

$$\frac{\mathrm{d}w}{\mathrm{d}t}=k_{s}\left(w_{\infty }-w\right)$$

$$\frac{t}{w}=A+Bt$$

### 2.10. Conductivity Tests

The conductivity of the gel was characterized by the conventional voltametric curve method and the voltametric curves of the samples were measured using a Keithley 2410 digital source meter (Keithley, Cleveland, OH, USA).

## 3. Results and Discussion

### 3.1. Network Structure and Swelling Properties

**Figure 1:**
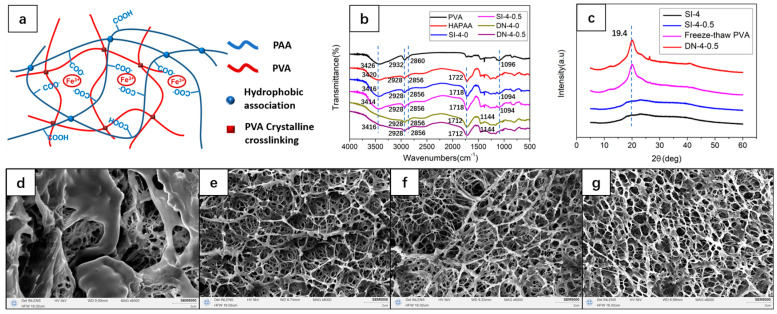
(**a**) Schematic representation of a multi-physically cross-linked hydrogel. (**b**) FTIR spectroscopy. (**c**) XRD patterns of the gels. SEM images of the gels: (**d**) HAPAA (**e**) DN-2-0.25 (**f**) DN-2-1 (**g**) DN-4-1.

According to the above preparation process, a series of multi-physically cross-linked hydrogels with different PVA and ferric chloride contents were obtained. The schematic representation of the hydrogel is shown in Figure 1a. Since acrylic acid was used as the hydrophilic monomer, the hydrophobic cross-linked network contained a large number of carboxylate ions and carboxyl groups, which formed a physically cross-linked structure with Fe^3+^ by ionic complexation and, together with the hydrophobic cross-linked network, formed the first physically cross-linked network of the dual network. After the freeze–thaw treatment, the PVA chains formed clusters of crystalline regions between the hydroxyl groups, which acted as physical cross-linking points and formed the second physical cross-linking network of the dual network. At the same time, the extensive hydroxyl groups on the PVA chains formed extensive hydrogen bonds with the HAPAA network, resulting in multiple extensive physical crosslinking networks, which contributed to the stability of the hydrogel network structure and the improvement of the mechanical properties. The FTIR spectroscopy of dried gel samples, as shown in Figure 1b, highlights that PVA showed the methylene asymmetric stretching vibration peak and the methylene symmetric stretching vibration peak from the main chain at 2932 and 2860 cm^−1^, respectively. HAPAA also showed these two peaks at similar positions, and the composite gels showed these two peaks and remained consistent with the position of HAPAA. The C=O stretching vibration peak from the carboxyl group appeared at 1722 cm^−1^ in HAPAA, which was not present in PVA. The peaks of composite gels SI-4-0 and SI-4-0.5 were redshifted to 1718 cm^−1^, and the peaks of DN-4-0 and DN-4-0.5 were redshifted to 1712 cm^−1^, which may be caused by the hydrogen-bonding interaction between the carboxyl group and hydroxyl group. The C-OH stretching vibration peak of the hydroxyl group appeared at 1096 cm^−1^ in PVA. In contrast, here, the peak shape of HAPAA was significantly different. The peaks of SI-4-0 and SI-4-0.5 were similar to those of PVA. The position had a slight red shift (from 1096 to 1094 cm^−1^), which may be caused by the hydrogen-bonding interaction. However, DN-4-0 and DN-4-0.5 did not show significant peaks here, possibly due to the formation of crystalline clusters of the hydroxyl groups after freeze–thaw treatment. DN-4-0 and DN-4-0.5 showed significant C-C-C stretching vibration peaks related to crystallization at 1144 cm^−1^, indicating an increase in crystallinity of the sample after freeze–thaw treatment. The broad peak between 3200 and 3600 cm^−1^ was the -OH stretching vibration peak. Compared with PVA and HAPAA, the peak of SI-4-0 and SI-4-0.5 had a red shift (from 3426 to 3414 cm^−1^), while DN-4-0 showed a broad blunt peak, which may be due to the reduction of free hydroxyl after freeze–thaw treatment. In contrast, DN-4-0.5 showed an insignificant peak at 3416 cm^−1^, which may be due to the interference of iron ions on the association of hydroxyl groups. The formation of a crystalline PVA was confirmed by XRD patterns as shown in Figure 1c. The SI-4 sample without freeze–thaw treatment did not show the crystalline characteristic diffraction peak of PVA, and the curve of the SI-4-0.5 sample with the addition of ferric chloride also showed no significant change, while after freeze–thaw treatment, the DN-4-0.5 sample showed an obvious characteristic diffraction peak of PVA crystals at 2θ = 19.4°, which was consistent with the freeze–thawed PVA samples, proving the formation of PVA crystalline structure in the composite gel. Changes in the morphological structure of the gel surface can be observed by SEM. SEM images of the samples after lyophilisation and platinum spray treatment are shown in Figure 1c–f. These samples all show spongy porous structures. In contrast, the pores of the HAPAA samples without PVA tended to collapse during the dynamic drying treatment, while DN-2-0.25 and DN-2-1 with added PVA had a more uniform macroporous over the small pore structure, probably because PVA played a supporting role during the freezing process and prevented the collapse of the pores compared to the insignificant effect of the ferric chloride content. In contrast, DN-4-1 with higher PVA content maintained a more homogeneous and detailed pore structure, and this structural change may be beneficial to improve the stability and mechanical properties.

**Figure 2:**
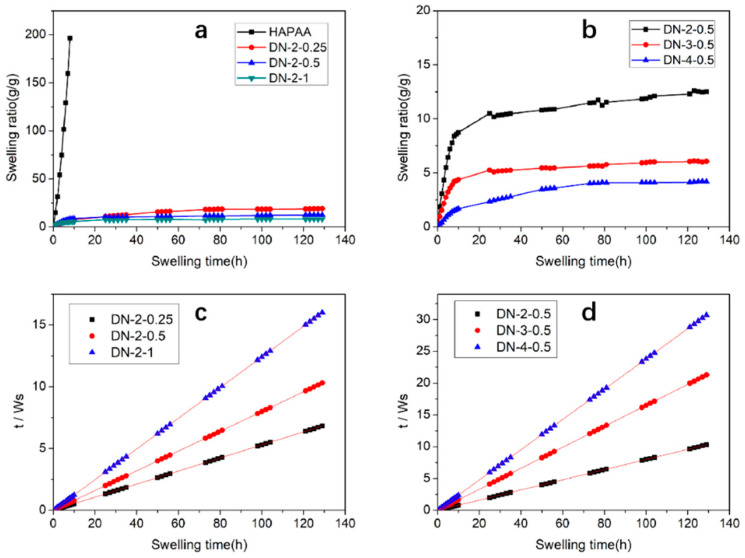
The swelling curves for (**a**) HAPAA, DN-2-Y and (**b**) DN-X-0.5 gels. Second-order fitting lines of (**c**) DN-2-Y and (**d**) DN-X-0.5 gels.

The swelling tests confirmed that the multiple physically cross-linked hydrogels exhibited good swelling stability upon immersion in water, and the swelling curves of the dry gels in DI water, as shown in Figure 2. The HAPAA gel with a purely hydrophobic cross-linked network showed poor dimensional stability, and when swelling occurred rapidly in water with a swelling ratio of more than 180 times and dissolving after more than 9 h of immersion, it was unable to maintain the complete hydrogel morphology. In contrast, the DN gels showed good swelling stability and their swelling ratios were significantly reduced, and the stable gel structures were maintained even after 6 days of immersion. Additionally, the equilibrium swelling ratio decreased significantly with increasing Fe^3+^ and PVA content, respectively, in which the DN-4-0.5 sample reached only 4.2 times the swelling ratio, which was less than the DN-4-0.5 hydrogel itself when it was synthesized, because on the one hand, the PVA crystalline network that formed by freezing was limited the swelling of the whole hydrogel network, and on the other hand, Fe^3+^ and carboxylate ions rearranged in water to form a more compact cross-linked network structure, leading to an increase in the gel network density. This rearrangement process took a long time, so that the composite gel did not reach an equilibrium dissolved state until about 100 h. During the initial swelling period, the difference in osmotic pressure between the inside and outside of the hydrogel promoted the rapid entry of water molecules into the hydrogel, resulting in a significant swelling rate for all hydrogel samples during the first few hours. Then, the differential pressure between the inside and outside of the hydrogel decreased and the swelling rate of the hydrogel began to slow. Figure 2c,d show the linear regression of the swelling curve obtained by using Schott’s second order model. The pure HAPAA gel dissolved after prolonged immersion; it did not have a swelling equilibrium state. The composite hydrogels with different components showed good linear relationships in Schott’s second order model, demonstrating that the swelling kinetics of the hydrogels followed second order kinetics.

### 3.2. Mechanical Properties

**Figure 3:**
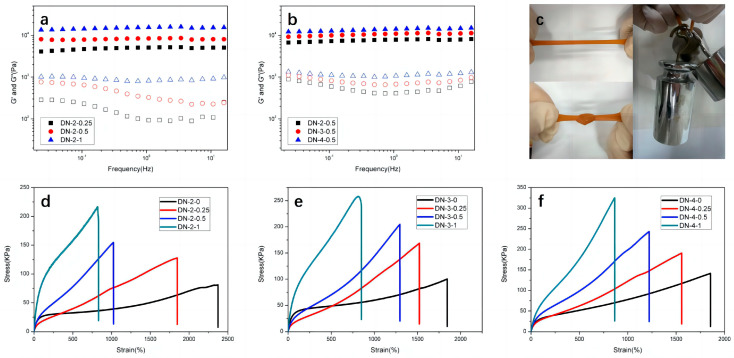
G′ and G″ of (**a**) DN-2-Y and (**b**) DN-X-0.5 hydrogels. (**c**) Images of DN-4-1 hydrogel stretching, stretching after being knotted and hanging three weights of 2 kg, 1 kg, 500 g. Stress–strain curves for (**d**) DN-2-Y (**e**) DN-3-Y and (**f**) DN-4-Y hydrogels.

Changes in components and structure also led to changes in the mechanical properties. The energy storage modulus G′ and the loss modulus G″ for the hydrogels with different Fe^3+^ and PVA contents are shown in Figure 3a,b, respectively. It was observed from the figures that the energy storage modulus G′ was always much higher than the loss modulus G″, and remained smooth in the frequency range tested, which was an important feature for the formation of the hydrogel cross-linked network. Figure 3a showed the DN hydrogels with constant PVA content and varying ferric chloride content, where the energy storage modulus increased with increasing ferric chloride content. Figure 3b showed DN hydrogels with constant ferric chloride content and varying PVA content, where the energy storage modulus also increased with increasing PVA content. This indicated that the addition of both PVA and ferric chloride acted to enhance the hydrogel. The synthesized DN-4-1 hydrogel exhibited high strength, as shown in Figure 3c. The appearance of the hydrogel was opaque like brown rubber because the addition of ferric chloride made the hydrogel brown and the freeze–thaw crystallization of PVA significantly reduce the transparency. In addition, the hydrogel still exhibited good tensile elongation and bending knotting ability. The tensile stress–strain curves of the hydrogels were shown in Figure 3d–f. Among the figures, Figure 3d showed the tensile stress–strain curves of DN-2 series hydrogels with constant PVA content and gradually increasing ferric chloride content, and the tensile strength of the hydrogels increased significantly with increasing ferric chloride content and the elongation decreased, among which the strength of DN-2-1 gel reached 242 kPa. The addition of Fe^3+^ reduced the mutual exclusion between the carboxyl groups on polyacrylic acid and the anionic surfactant SDBS, which can play a certain stabilizing effect on the hydrophobic association micro-region and, therefore, macroscopically showed an increased strength, where the strength of DN-4-1 hydrogel reached 367 kPa. With increasing PVA content, the composite hydrogels also showed an increase in strength and a lesser decrease in elongation, and the limitation of the PVA crystalline cross-linked network on gel elongation was lower, probably because of the extensive hydrogen-bonding interaction between the carboxyl groups of polyacrylic acid chains and the hydroxyl groups of the PVA chains, which reduced the formation of PVA crystalline clusters. Therefore, macroscopically, there was no significant reduction in elongation, which had a positive significance for the preparation of hydrogels with both high strength and high elongation.

**Figure 4:**
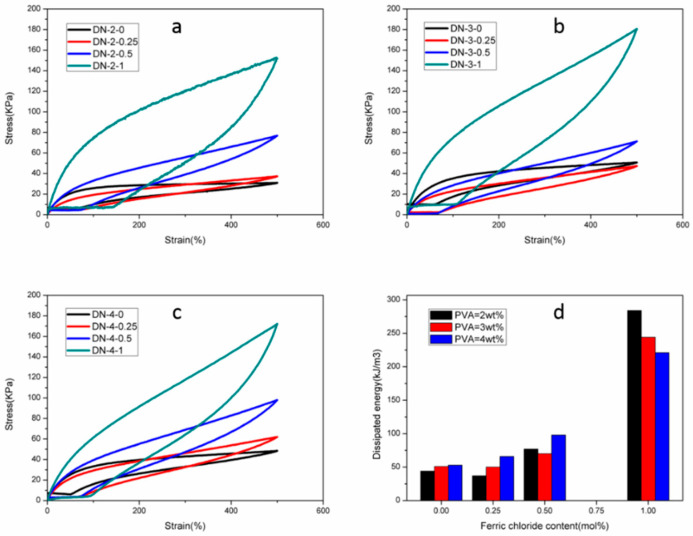
Cyclic stretching curves of: (**a**) DN-2-Y (**b**) DN-3-Y hydrogels and (**c**) DN-4-Y hydrogels. (**d**) Dissipated energies calculated from the hysteresis loops.

Energy dissipation capability is an important index to evaluate the mechanical properties of physically cross-linked hydrogels. Hydrophobic association, hydrogen-bonding interaction and ionic complexation are all physical cross-linking interactions that can effectively dissipate the energy during the stretching process of the hydrogels, so the multiple physically cross-linked hydrogels synthesized in this study had good energy dissipation capability. This was verified in the cyclic stretching tests of the hydrogel at 500% elongation, and the effect of PVA and ferric chloride content on the energy dissipation capability was also investigated, as shown in Figure 4. The energy dissipation of the hydrogels all increased significantly with the increase in Fe^3+^ content, because the increase in Fe^3+^ can provide more ionic complexes physical cross-linking points, which were subjected to destruction and stress consumption when subjected to external forces, thus helping to improve the energy dissipation in the stretching process, which increased to about six times the initial energy dissipation at 500% elongation compared to the sample without Fe^3+^. In contrast, the change in PVA content showed less effect on the energy dissipation, probably due to the high stability of the PVA cluster crystalline region, which was difficult to disrupt at this strain. Even at a higher Fe^3+^ content, the dissipation energy of the hydrogel decreased with increasing PVA content, probably because after the formation of the dual network structure; the PVA crystalline cross-linked network hindered the movement of the polyacrylic acid chains, which hindered the disruption and reorganization of the cross-linking structure of Fe^3+^ and carboxylate ions, so that the dissipation energy decreased instead.

**Figure 5:**
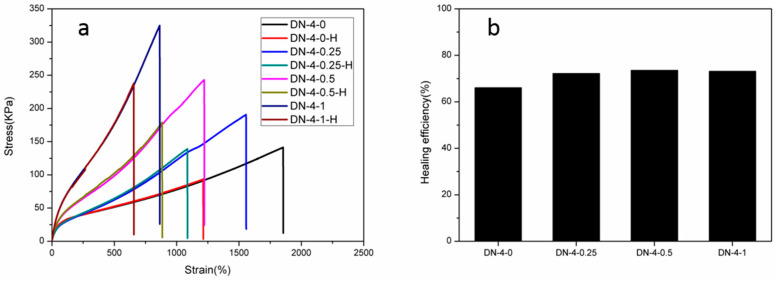
(**a**) Tensile stress–stain curves of the original and healed hydrogels. (**b**) Healing efficiencies calculated from tensile strength.

The DN-4 series hydrogels showed good overall mechanical properties in the previous tests and were therefore selected for further testing of the self-healing properties. In order to assess the self-healing properties of the hydrogels, the gel samples were cut into two sections and then placed in a sample bag with the two sections touching each other, and were then left at room temperature for two days before being subjected to a uniaxial tensile test, and the self-healing ratio of the hydrogels was evaluated from the tensile strength data. As shown in Figure 5, the DN-4-0 hydrogel without ferric chloride showed good self-healing ability with a fracture stress of 93.5 kPa, approximately 66% of the original sample. The good healing ratio was attributed to the rearrangement formation of the hydrophobic association region and the formation of the extensive hydrogen-bonding interactions between PAA and PVA, which contributed to the rapid self-healing of the hydrogels. In contrast, the self-healing properties of the hydrogels containing ferric chloride were further improved, reaching 72.3%, 73.6% and 73.2% for DN-4-0.25, DN-4-0.5 and DN-4-1 gels, respectively, because the ferric ion complexes that were formed by the addition of ferric chloride rearranged to form new cross-linked structures after disruption, which promoted the self-healing of the hydrogels. Therefore, it was experimentally confirmed that hydrogels with both high-strength and good self-healing properties could be obtained by synthesizing suitable multiple physically cross-linked hydrogels. Compared with the gelatin/polyacrylic acid/tannic acid/aluminum chloride tetradic double-network physical crosslinking hydrogel sensor reported by Wei Feng et al. [38], the hydrogel synthesized in this work had a significant advantage in strength (367 > 85.4 kPa), but a lower healing ratio (73.2 < 88.3%). Compared with the polyacrylic acid/polyaniline dual-network-conductive hydrogel sensor reported by Guihua Yu et al. [22], the strength of the gel synthesized in this work was significantly lower (367 < 910 kPa), but because polyaniline itself had no significant self-healing ability, the healing rate in this work was higher (73.2% > 65.9%). Therefore, the multi-physically cross-linked hydrogels synthesized in this work have achieved a certain balance between high-strength and self-healing ability, but the contradiction between strength and healing efficiency of self-healing hydrogels was not completely resolved.

### 3.3. Conductive Sensing Tests

**Figure 6:**
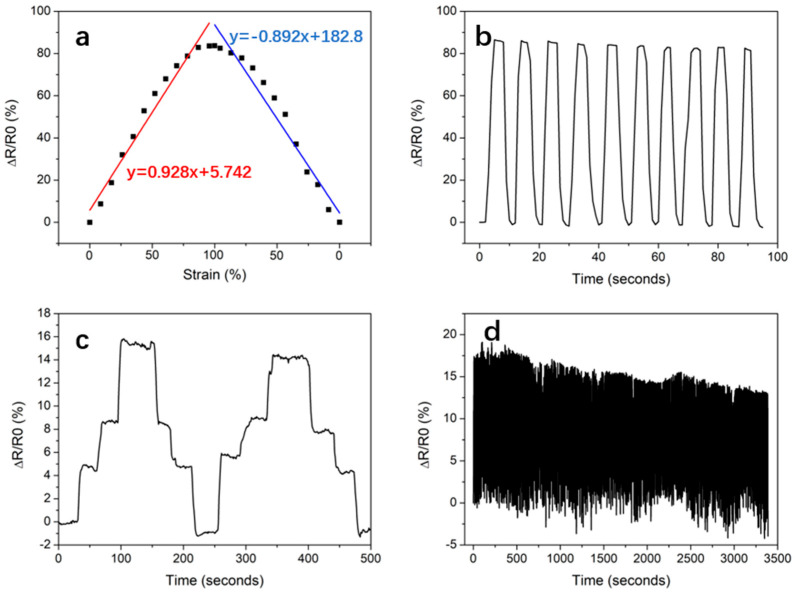
(**a**) The relative change in resistance of the DN-4-1 hydrogel changes with stretching; (**b**) repeated stretching to 100%; (**c**) step changes with finger flexion at different angles; (**d**) continuous repetition of 20% cyclic stretching.

DN-4-1 hydrogel with the highest mechanical strength and good self-healing property was selected for the conductive sensing tests. In the polymerization process of this hydrogel, various ions such as H^+^, Cl^−^ and Fe^3+^ were introduced, so the composite hydrogel has a certain ionic conductivity, and its conductivity is about 0.076 s/m at room temperature by voltammetry using a digital source meter, and the hydrogel had the potential to be used as a flexible sensor because it can clearly reflect the change in test current when subjected to an external force. The change in relative resistance with strain is shown in Figure 6a. The hydrogel was uniformly stretched to 100% strain and the hydrogel sample was deformed during the stretching process, the length increased and the cross-sectional area decreased, making ion transport more difficult, resulting in an increase in resistance. Then, the hydrogel gradually returned to its original length and the resistance gradually decreased back to close to its initial value, with its relative resistance changing up to 87% throughout the whole process, showing a sensitive response. Linear fits to the test data showed that the GF (gauge factor) = 0.928 during stretching and 0.892 during shrinking; the difference between the two values may be due to some hysteresis in the strain response of the hydrogel. The relative resistance changed stably when the hydrogel was cyclically stretched to 100% strain several times within a short period of time, as shown in Figure 6b. Then, the applicability of this hydrogel for the finger joint movements sensor was investigated. The hydrogel was attached to the researcher’s finger joint using acrylic tape, and the bending movements of the finger joint at different angles were recorded, as shown in Figure 6c. According to different degrees of bending, the relative resistance showed a step change curve, and the test curve characteristics were clear and obvious without any apparent delay. The relative resistance change at the maximum degree of bending was approximately equal to 20% elongation of the hydrogel. Repeated cyclic strain tests were performed more than 100 times at 20% strain, as shown in Figure 6d, and the change curve showed good repeatability and stability, indicating that this multi-physically cross-linked hydrogel had a good strain sensor effect.

## 4. Conclusions

In conclusion, a composite hydrogel with multiple physically cross-linked structures, including hydrophobic association, hydrogen bonding and ionic complexation, was successfully prepared in this study. Both PVA and ferric chloride contribute to the mechanical strength of the composite hydrogel, but ferric chloride causes a significant decrease in the elongation of the hydrogel, while the effect of PVA on the elongation is relatively small. The energy dissipation capacity of the physical hydrogels increased significantly with the increasing ferric chloride content because the increase in Fe^3+^ could provide more physical cross-linking points and contribute to the energy dissipation during the elongation process; the increase in PVA had less effect on the energy dissipation, and even at higher Fe^3+^ content, the energy dissipation decreased with increasing PVA content, probably because PVA hinders the movement of the polyacrylic acid chain segments, thus hindering the disruption and reorganization of the interaction structure of Fe^3+^ and carboxyl ions. The hydrogel exhibits both good self-healing and mechanical properties, and the application of this composite hydrogel as a conductive sensor was tested in this study, which is an important reference for the preparation of high-performance gel sensors with both high-strength and self-healing properties.

## Figures and Tables

**Table 1:** Feed composition for preparation of hydrogels.

Gels	AA (g)	SMA (g)	SDBS (g)	PVA (g)	FeCl_3_·6H_2_O (g)	KPS (g)	H_2_O (g)
HAPAA	3	0.22	0.45	0	0	0.01	11.32
PAA-2-0.25	3	0.22	0.45	0.3	0.028	0.01	11
PAA-2-0.5	3	0.22	0.45	0.3	0.056	0.01	10.96
PAA-2-1	3	0.22	0.45	0.3	0.112	0.01	10.91
PAA-3-0.25	3	0.22	0.45	0.45	0.028	0.01	10.85
PAA-3-0.5	3	0.22	0.45	0.45	0.056	0.01	10.82
PAA-3-1	3	0.22	0.45	0.45	0.112	0.01	10.77
PAA-4-0.25	3	0.22	0.45	0.6	0.028	0.01	10.7
PAA-4-0.5	3	0.22	0.45	0.6	0.056	0.01	10.67
PAA-4-1	3	0.22	0.45	0.6	0.112	0.01	10.62

## Data Availability

Not applicable.

## References

[1] Emam H.E., Shaheen T.I. (2022). Design of a dual pH and temperature responsive hydrogel based on esterified cellulose nanocrystals for potential drug release. Carbohydr. Polym..

[2] Zu S., Wang Z., Zhang S., Guo Y., Chen C., Zhang Q., Wang Z., Liu T., Liu Q., Zhang Z. (2022). A bioinspired 4D printed hydrogel capsule for smart controlled drug release. Mater. Today Chem..

[3] Bai Z., Wang X., Zheng M., Yue O., Huang M., Zou X., Cui B., Xie L., Dong S., Shang J. (2023). Mechanically Robust and Transparent Organohydrogel-Based E-Skin Nanoengineered from Natural Skin. Adv. Funct. Mater..

[4] Bian S., Hao L., Qiu X., Wu J., Chang H., Kuang G.-M., Zhang S., Hu X., Dai Y., Zhou Z. (2022). An Injectable Rapid-Adhesion and Anti-Swelling Adhesive Hydrogel for Hemostasis and Wound Sealing. Adv. Funct. Mater..

[5] Huang X., Huang J., Yang D., Wu P. (2021). A Multi-Scale Structural Engineering Strategy for High-Performance MXene Hydrogel Supercapacitor Electrode. Adv. Sci..

[6] Lu H., Li M., Wang X., Wang Z., Pi M., Cui W., Ran R. (2022). Recyclable physical hydrogels as durable and efficient solar-driven evaporators. Chem. Eng. J..

[7] Su X., Hao D., Sun M., Wei T., Xu D., Ai X., Guo X., Zhao T., Jiang L. (2022). Nature Sunflower Stalk Pith with Zwitterionic Hydrogel Coating for Highly Efficient and Sustainable Solar Evaporation. Adv. Funct. Mater..

[8] Khan F., Atif M., Haseen M., Kamal S., Khan M.S., Shahid S., Nami S.A.A. (2022). Synthesis, classification and properties of hydrogels: Their applications in drug delivery and agriculture. J. Mater. Chem. B.

[9] Fu D., Huang G., Xie Y., Zheng M., Feng J., Kan K., Shen J. (2023). Novel Uracil-Functionalized Poly(ionic liquid) Hydrogel: Highly Stretchable and Sensitive as a Direct Wearable Ionic Skin for Human Motion Detection. ACS Appl. Mater. Interfaces.

[10] Kim Y., Yuk H., Zhao R., Chester S.A., Zhao X. (2018). Printing ferromagnetic domains for untethered fast-transforming soft materials. Nature.

[11] Wu H., Wang M., Wu W., Bai D., Liang Y., Hu S., Yu W., He P., Zhang J. (2023). Ionic liquid–polymer thermochromic electrolytes with a wide and tunable LCST for application in multi-stimuli-responsive optical modulation. J. Mater. Chem. A.

[12] Liang J., Li B., Gai X., Li N., Wang J., Zhang Y., Zhou Q., Sun Y. (2023). Photochromic/electrochromic strain sensor with a fast and reversible light-printing ability. J. Mater. Chem. C.

[13] Liu D., Huyan C., Wang Z., Guo Z., Zhang X., Torun H., Mulvihill D., Xu B.B., Chen F. (2023). Conductive polymer based hydrogels and their application in wearable sensors: A review. Mater. Horiz..

[14] Su G., Cao J., Zhang X., Zhang Y., Yin S., Jia L., Guo Q., Zhang X., Zhang J., Zhou T. (2020). Human-tissue-inspired anti-fatigue-fracture hydrogel for a sensitive wide-range human–machine interface. J. Mater. Chem. A.

[15] Zhang Y., Lu H., Li M., Yan B., Ran R. (2021). Near-Infrared Laser “Weldable” Hydrogen-Bonded Hydrogel Sensor Based on Photothermal Gel–Sol Transition. ACS Sustain. Chem. Eng..

[16] Su G., Zhang Y., Zhang X., Feng J., Cao J., Zhang X., Zhou T. (2022). Soft yet Tough: A Mechanically and Functionally Tissue-like Organohydrogel for Sensitive Soft Electronics. Chem. Mater..

[17] Liu S., Chen Y., Feng J., Peng J., Zhou Y., Zhao Y., Zhao Y., Lu Z., Sun M., Wu C. (2023). A mechanically soft-tissue-like organohydrogel with multi-functionalities for sensitive soft ionotronics. Chem. Eng. J..

[18] Dong X., Guo X., Liu Q., Zhao Y., Qi H., Zhai W. (2022). Strong and Tough Conductive Organo-Hydrogels via Freeze-Casting Assisted Solution Substitution. Adv. Funct. Mater..

[19] Ji D., Park J.M., Oh M.S., Nguyen T.L., Shin H., Kim J.S., Kim D., Park H.S., Kim J. (2022). Superstrong, superstiff, and conductive alginate hydrogels. Nat. Commun..

[20] Wu Y., Xing W., Wen J., Wu Z., Zhang Y., Zhang H., Wu H., Yao H., Xue H., Gao J. (2023). Mixed solvent exchange enabled high-performance polymeric gels. Polymer.

[21] Wu P., Qin Z., Dassanayake R., Sun Z., Cao M., Fu K., Zhou Y., Liu Y. (2023). Antimicrobial MXene-based conductive alginate hydrogels as flexible electronics. Chem. Eng. J..

[22] Su G., Yin S., Guo Y., Zhao F., Guo Q., Zhang X., Zhou T., Yu G. (2021). Balancing the mechanical, electronic, and self-healing properties in conductive self-healing hydrogel for wearable sensor applications. Mater. Horiz..

[23] Zhang Y., Chen K., Li Y., Lan J., Yan B., Shi L., Ran R. (2019). High-Strength, Self-Healable, Temperature-Sensitive, MXene-Containing Composite Hydrogel as a Smart Compression Sensor. ACS Appl. Mater. Interfaces.

[24] Shen K., Liu Z., Xie R., Zhang Y., Yang Y., Zhao X., Zhang Y., Yang A., Cheng Y. (2023). Nanocomposite conductive hydrogels with Robust elasticity and multifunctional responsiveness for flexible sensing and wound monitoring. Mater. Horiz..

[25] Li G., Li C., Li G., Yu D., Song Z., Wang H., Liu X., Liu H., Liu W. (2022). Development of Conductive Hydrogels for Fabricating Flexible Strain Sensors. Small.

[26] Yu J., Wang K., Fan C., Zhao X., Gao J., Jing W., Zhang X., Li J., Li Y., Yang J. (2021). An Ultrasoft Self-Fused Supramolecular Polymer Hydrogel for Completely Preventing Postoperative Tissue Adhesion. Adv. Mater..

[27] Shi X., Ma L., Li Y., Shi Z., Wei Q., Ma G., Zhang W., Guo Y., Wu P., Hu Z. (2023). Double Hydrogen-bonding Reinforced High-Performance Supramolecular Hydrogel Thermocell for Self-powered Sensing Remote-Controlled by Light. Adv. Funct. Mater..

[28] Hu Y., Cui Y., Que X., Zhang Z., Peng J., Li J., Zhai M. (2022). Super Adhesive MXene-based Nanocomposite Hydrogel with Self-Healable and Conductivity Properties via Radiation Synthesis. Adv. Eng. Mater..

[29] Huang S., Hou L., Li T., Jiao Y., Wu P. (2022). Antifreezing Hydrogel Electrolyte with Ternary Hydrogen Bonding for High-Performance Zinc-Ion Batteries. Adv. Mater..

[30] Zhu H., Dai W., Wang L., Yao C., Wang C., Gu B., Li D., He J. (2022). Electroactive Oxidized Alginate/Gelatin/MXene (Ti3C2Tx) Composite Hydrogel with Improved Biocompatibility and Self-Healing Property. Polymers.

[31] Cai S., Niu B., Ma X., Wan S., He X. (2022). High strength, recyclable, anti-swelling and shape-memory hydrogels based on crystal microphase crosslinking and their application as flexible sensor. Chem. Eng. J..

[32] Hao X.P., Zhang C.W., Zhang X.N., Hou L.X., Hu J., Dickey M.D., Zheng Q., Wu Z.L. (2022). Healable, Recyclable, and Multifunctional Soft Electronics Based on Biopolymer Hydrogel and Patterned Liquid Metal. Small.

[33] Li L., Li W., Wang X., Zou X., Zheng S., Liu Z., Li Q., Xia Q., Yan F. (2022). Ultra-Tough and Recyclable Ionogels Constructed by Coordinated Supramolecular Solvents. Angew. Chem. Int. Ed..

[34] Shi C., Yang F., Hu L., Wang H., Wang Y., Wang Z., Pan S., Chen J. (2022). Construction of polysaccharide based physically crosslinked double-network antibacterial hydrogel. Mater. Lett..

[35] Ailincai D., Turin Moleavin I.-A., Sarghi A., Fifere A., Dumbrava O., Pinteala M., Balan G.G., Rosca I. (2023). New Hydrogels Nanocomposites Based on Chitosan, 2-Formylphenylboronic Acid, and ZnO Nanoparticles as Promising Disinfectants for Duodenoscopes Reprocessing. Polymers.

[36] Jiang L., Huang X., Tian C., Zhong Y., Yan M., Miao C., Wu T., Zhou X. (2023). Preparation and Characterization of Porous Cellulose Acetate Nanofiber Hydrogels. Gels.

[37] Chen J., Wang X., Dao L., Liu L., Yang Y., Liu J., Wu S., Cheng Y., Pang J. (2022). A conductive bio-hydrogel with high conductivity and mechanical strength via physical filling of electrospinning polyaniline fibers. Colloids Surf. A.

[38] Bai H., Zhang Z., Huo Y., Shen Y., Qin M., Feng W. (2022). Tetradic double-network physical crosslinking hydrogels with synergistic high stretchable, self-healing, adhesive, and strain-sensitive properties. J. Mater. Sci. Technol..

[39] Matsuda T., Kawakami R., Namba R., Nakajima T., Gong J.P. (2019). Mechanoresponsive self-growing hydrogels inspired by muscle training. Science.

[40] Madduma-Bandarage U.S.K., Madihally S.V. (2021). Synthetic hydrogels: Synthesis, novel trends, and applications. J. Appl. Polym. Sci..

[41] Hong L., Liu L., Zhang Z., Song J., Li S., Chen K., Gao G., Wang Y. (2022). Tough and self-healing hydrogels based on transient crosslinking by nanoparticles. Soft Matter.

[42] Rahmani P., Shojaei A. (2022). Developing tough terpolymer hydrogel with outstanding swelling ability by hydrophobic association cross-linking. Polymer.

[43] Zhang M., Yang Y., Li M., Shang Q., Xie R., Yu J., Shen K., Zhang Y., Cheng Y. (2023). Toughening Double-Network Hydrogels by Polyelectrolytes. Adv. Mater..

[44] Abolpour Moshizi S., Moradi H., Wu S., Han Z.J., Razmjou A., Asadnia M. (2022). Biomimetic Ultraflexible Piezoresistive Flow Sensor Based on Graphene Nanosheets and PVA Hydrogel. Adv. Mater. Technol..

[45] Adelnia H., Ensandoost R., Shebbrin Moonshi S., Gavgani J.N., Vasafi E.I., Ta H.T. (2022). Freeze/thawed polyvinyl alcohol hydrogels: Present, past and future. Eur. Polym. J..

[46] Karimzadeh Z., Mahmoudpour M., Rahimpour E., Jouyban A. (2022). Nanomaterial based PVA nanocomposite hydrogels for biomedical sensing: Advances toward designing the ideal flexible/wearable nanoprobes. Adv. Colloid Interface Sci..

[47] Zhu X., Ji C., Meng Q., Mi H., Yang Q., Li Z., Yang N., Qiu J. (2022). Freeze-Tolerant Hydrogel Electrolyte with High Strength for Stable Operation of Flexible Zinc-Ion Hybrid Supercapacitors. Small.

[48] Janarthanan G., Noh I. (2021). Recent trends in metal ion based hydrogel biomaterials for tissue engineering and other biomedical applications. J. Mater. Sci. Technol..

[49] Cao J., Wang Y., He C., Kang Y., Zhou J. (2020). Ionically crosslinked chitosan/poly(acrylic acid) hydrogels with high strength, toughness and antifreezing capability. Carbohydr. Polym..

